# Standardized Myocardial T1 and T2 Relaxation Times: Defining Age- and Comorbidity-Adjusted Reference Values for Improved CMR-Based Tissue Characterization

**DOI:** 10.3390/jcm14176198

**Published:** 2025-09-02

**Authors:** Mukaram Rana, Vitali Koch, Simon Martin, Thomas Vogl, Marco M. Ochs, David M. Leistner, Sebastian M. Haberkorn

**Affiliations:** 1 Department of Cardiology and Angiology, University Hospital, Goethe University Frankfurt, 60590 Frankfurt, Germany; 2 Department of Radiology, University Hospital, Goethe University Frankfurt, 60590 Frankfurt, Germany; 3 German Center of Cardiovascular Research (DZHK), Partner Site Rhine/Main, 60590 Frankfurt, Germany; 4 German Center of Cardiovascular Science (DZHK), Cardiopulmonary Institute (CPI), 60590 Frankfurt, Germany

**Keywords:** T1 mapping, cardiac MRI, T2 mapping, MOLLI, GRASE, myocardial tissue characterization, myocardial relaxation times, quantitative imaging, comorbidities, age, sex, gender

## Abstract

**Background:** This study aims to establish standardized reference values for myocardial T1 and T2 relaxation times in a clinically and imaging-defined real-world patient cohort, evaluating their variability in relation to age, sex, and comorbidities. By identifying key physiological and pathological influences, this investigation seeks to enhance CMR-based myocardial mapping for improved differentiation between normal and pathological myocardial conditions. **Methods:** This retrospective observational study analyzed T1 and T2 relaxation times using CMR at 1.5 Tesla in a cohort of 491 subjects. T1 and T2 times were measured using MOLLI and GRASE sequences, and statistical analyses assessed intra- and interindividual variations, including the influence of age, sex, and comorbidities, to establish reference values and improve myocardial tissue characterization. **Results:** T1 and T2 relaxation times were analyzed in 291 and 200 participants, respectively. The mean global T1 time was 1004.7 ± 49.8 ms, with no significant differences between age groups (*p* = 0.81) or sexes (*p* = 0.58). However, atrial fibrillation (AF) and mitral regurgitation (MR) were associated with significantly prolonged T1 times (*p* < 0.05). The mean global T2 time was 67.4 ± 8.6 ms, with age-related prolongation (*p* < 0.05), but no sex differences (*p* = 0.46). Comorbidities did not significantly influence T2 times, except for NYHA Class III–IV patients, who exhibited prolonged T2 values (*p* < 0.05). **Conclusions:** Standardized T1 and T2 reference values are essential to improve diagnostic accuracy and risk stratification in CMR-based myocardial tissue characterization. Future research should focus on multicenter validation, AI-driven analysis, and the development of age- and comorbidity-adjusted normative databases to enhance individualized cardiovascular care.

## 1. Introduction

Cardiovascular diseases (CVD) remain the leading cause of mortality worldwide [1]. The accurate diagnosis and risk stratification of CVDs are crucial for effective clinical management, particularly in the early identification of myocardial pathology. Recent advances in non-invasive imaging modalities, such as cardiac magnetic resonance imaging (CMR), have significantly improved the assessment of myocardial tissue composition and function [2,3]. Among these, T1 and T2 mapping techniques have emerged as powerful tools for myocardial tissue characterization, providing quantitative biomarkers for detecting pathological changes such as fibrosis, edema, and infiltration [4,5,6].

CMR offers a high degree of sensitivity and specificity for diagnosing cardiomyopathies and myocarditis and is considered the gold standard for myocardial tissue characterization [7]. Specifically, late gadolinium enhancement (LGE) imaging enables the detection of focal myocardial fibrosis, while T1 and T2 mapping techniques allow for the quantification of diffuse myocardial changes [4]. Despite their diagnostic potential, T1 and T2 relaxation times are influenced by multiple physiological and pathological factors, including age, sex, ethnicity, and comorbidities such as chronic kidney disease and diabetes mellitus [8]. These variables can significantly affect the interpretation of myocardial relaxation times, highlighting the need for standardized reference values that account for these confounding factors [9].

Currently, there is a lack of universally accepted reference values for T1 and T2 relaxation times in the myocardium of a representative real-world patient population [10]. Without well-established normative data, the clinical applicability of myocardial mapping remains limited, particularly in distinguishing physiological variations from pathological changes. To address this gap, the present study retrospectively analyzes a real-world cohort that includes both individuals without known cardiac pathology and patients with various comorbidities. The inclusion of subjects across a spectrum of cardiovascular risk profiles—including differences in age, sex, and comorbid conditions—allows for a systematic evaluation of how these factors influence myocardial T1 and T2 relaxation times.

The aim is to investigate whether such demographic and clinical variables significantly impact relaxation values and therefore need to be considered when establishing standardized reference ranges. Currently, these influencing factors are not routinely accounted for in proposed standard values, which may limit the diagnostic accuracy of CMR-based myocardial tissue characterization in diverse clinical populations.

T1 and T2 relaxation times are quantitative markers that change dynamically in response to myocardial pathology, including myocardial infarction, myocarditis, and amyloidosis. These parameters can be measured without contrast agents, using the Modified Look-Locker Inversion Recovery (MOLLI) sequence for T1 mapping and a gradient and spin-echo (GRASE)-based sequence for T2 mapping [10]. By evaluating myocardial relaxation times in a real-world patient population, this study will investigate intraindividual (regional) and interindividual variations in relation to age and sex. It is hypothesized that myocardial T1 and T2 relaxation times are independent of age and sex and that, in an age- and sex-matched cohort, there are no significant regional variations in myocardial relaxation times.

Advances in artificial intelligence (AI) have introduced novel approaches for automated myocardial mapping and segmentation, reducing interobserver variability and streamlining clinical workflows [9,11]. While this study focuses on conventional MOLLI and GRASE techniques, the integration of AI-based mapping into future protocols may facilitate the unification of measurements across different scanners and populations, thereby enhancing generalizability and clinical applicability.

This study aims to contribute to the refinement of myocardial mapping as a diagnostic tool by providing robust reference values that enhance its clinical applicability. Standardized relaxation time parameters could improve the differentiation between normal myocardial physiology and disease, ultimately advancing CMR-based diagnostics and patient management.

## 2. Materials and Methods

### 2.1. Study Design and Patient Population

This retrospective observational study was conducted at a university hospital as part of the registry study “Prognostic Significance of a Non-Invasive Myocardial Ischemia Test Using CMR with Regadenoson.” Patient data were retrospectively analyzed, and relevant clinical and imaging parameters were obtained from electronic medical records. The study was approved on 16 May 2018 by the local ethics committee (2018-65-RetroDEuA) and complied with the ethical principles outlined in the Declaration of Helsinki.

### 2.2. Inclusion Criteria

Prior to participation, all potential subjects received both oral and written explanations regarding the study’s purpose, methods, and potential risks. Informed consent was obtained before inclusion. The inclusion criteria were as follows:

- Age > 18 years;
- Full legal capacity to provide consent;
- Cardiovascular health defined as the absence of ischemia or myocardial scarring in CMR;
- Clinical indication for stress CMR, justifying the use of a myocardial ischemia test;
- Suitability for regadenoson administration, without contraindications such as severe obstructive pulmonary disease or acute heart failure.

### 2.3. Exclusion Criteria

Patients were excluded from the study if they met any of the following criteria:

- Presence of ferromagnetic implants, such as pacemakers, implantable cardioverter defibrillators (ICD), or vascular clips, posing a safety risk during CMR;
- Pregnancy or lactation, due to insufficient data on potential effects of the magnetic field;
- Claustrophobia preventing completion of the CMR examination;
- Poor image quality due to motion or technical artifacts;
- Withdrawal of consent or the development of contraindications during the examination.

### 2.4. Comorbidities

A total of 19 comorbidities were assessed for their potential influence on T1 and T2 relaxation times. These included New York Heart Association (NYHA) and Canadian Cardiovascular Society (CCS) classifications for heart failure and coronary artery disease, respectively. Coronary artery disease was further stratified into single-, double-, and triple-vessel disease. Valvular heart disease was categorized into stenosis and regurgitation, with a focus on aortic stenosis, aortic regurgitation, mitral stenosis, mitral regurgitation, and tricuspid regurgitation. The left ventricular ejection fraction (LVEF) was classified according to guidelines from the German Cardiac Society (DGK) and the European Society of Cardiology (ESC), with values > 55% considered normal and values < 55% indicating impaired function according to current guideline recommendations [4]. Other assessed comorbidities included chronic kidney disease, diastolic dysfunction, cardiomyopathy, atrial fibrillation, chronic obstructive pulmonary disease (COPD), hypertension, smoking, hyperlipidemia, diabetes mellitus, and obesity.

### 2.5. CMR Examination Protocol

CMR scans were performed using a 1.5 Tesla MRI scanner (Philips Achieva, Best, The Netherlands) at the university hospital’s cardiology department. Patients were placed in the supine position with a dedicated five-element receiver coil applied to the chest. The coil utilized SENSE technology for enhanced image quality. Vital parameters, including heart rate, blood pressure, and respiration, were continuously monitored using an MRI-compatible system. All CMR studies were conducted by trained radiology technicians under the supervision of an experienced cardiologist. Contrast agents and stress test medications were administered via a peripheral venous catheter. Prior to the procedure, patients were thoroughly informed about the examination process and provided written consent. The total scan time was approximately 45 min, including regional wall motion analysis and global cardiac function assessment using multiple pulse sequences.

### 2.6. Cardiac Function Analysis

Left ventricular systolic and diastolic function were assessed using cine-MRI. End-systolic and end-diastolic volumes, stroke volume, and LVEF were determined using Simpson’s disk summation method. Imaging was performed in four-chamber and short-axis views to analyze cardiac anatomy and function. Cine loops in three short-axis slices and four-chamber views were acquired using balanced steady-state free precession (bSSFP) cine-MRI (TR/TE = 2.9/1.5 ms, FA = 60, spatial resolution 8 × 1.5 × 1.5 mm, 35 phases per cardiac cycle, breath-hold technique). Diastolic dysfunction was defined according to established echocardiographic criteria (ASE/EACVI 2016), based on impaired relaxation patterns and/or elevated filling pressures. Specifically, parameters including an E/e′ ratio > 14, and a left atrial volume index > 34 mL/m^2^ were applied to categorize diastolic impairment [12].

#### 2.6.1. T1 Mapping–MOLLI

T1 relaxation times were measured using the Modified Look-Locker Inversion Recovery (MOLLI) sequence, a widely applied CMR technique for assessing myocardial tissue properties. MOLLI employs a series of inversion recovery pulses and Look-Locker readout steps to acquire multiple images at different inversion times. These data were used to generate T1 maps, providing insights into myocardial fibrosis, inflammation, and edema. T1 mapping was conducted with a 5(3)3-MOLLI sequence (5 acquisition heartbeats followed by 3 recovery heartbeats and a further 3 acquisition heartbeats) with an inversion time (TI) of 120 ms, a TI increment of 80 ms, and a spatial resolution of 2.6 × 2.2 × 8 mm^3^. The MOLLI protocol was optimized for cardiac applications to ensure motion robustness and high reproducibility.

#### 2.6.2. T2 Mapping–GRASE

T2 relaxation times were quantified using the gradient and spin-echo (GRASE) sequence, which integrates a turbo spin-echo (TSE) sequence with echo-planar imaging (EPI) as described previously [13]. In brief, a stack of 15 images with increasing echo times (TE) (10 ms inter-echo spacing) was acquired at end-diastole (repetition time (TR): 1 cardiac cycle, flip angle 90°, spatial resolution 2.6 × 2.6 × 10 mm^3^). This technique enables high-resolution myocardial imaging with an improved contrast-to-noise ratio and reduced motion artifacts. Respiratory motion was controlled using a navigator-gated approach to ensure data accuracy.

### 2.7. Image Analysis and Data Processing

T1 and T2 maps were analyzed using CVI42 software (Circle Cardiovascular Imaging Inc., Calgary, AB, Canada). Image segmentation was performed according to recommendations from the Society for Cardiovascular Magnetic Resonance (SCMR) and the European Association for Cardiovascular Imaging (EACVI). Left ventricular slices were divided into basal, mid-ventricular, and apical segments. Each region of interest (ROI) was manually outlined to exclude pericardial and blood pool contamination. Visual assessment of image quality was performed to exclude motion artifacts or errors in image registration. Outliers exceeding one standard deviation were re-evaluated for potential technical artifacts or measurement errors. Non-correctable artifacts led to case exclusion from statistical analysis.

### 2.8. Statistical Analysis

Statistical analyses were performed using IBM SPSS Statistics for Windows (Version 27.0, IBM Corp., Armonk, NY, USA). Continuous variables were reported as mean ± standard deviation. Categorical variables were expressed as absolute values and percentages. Normality was assessed using the Kolmogorov–Smirnov and Shapiro–Wilk tests. Group comparisons for continuous variables were conducted using independent and paired t-tests. Statistical significance was defined as a *p*-value < 0.05, with all analyses using a 95% confidence interval.

## 3. Results

### 3.1. Study Population and Clinical Characteristics

**Figure 1:**
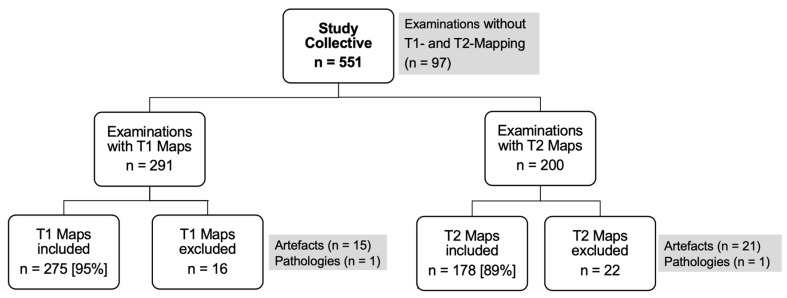
Study selection process.

Between 2015 and 2017, a total of 551 subjects were recruited for this study at the University Hospital. After applying the inclusion criteria, 491 participants (89%) were included in the final analysis (see Figure 1). All subjects underwent T1 relaxation time measurements using the MOLLI method and/or T2 relaxation time measurements using the GRASE method for clinical indications.

For T1 mapping, 291 examinations were performed, of which 275 (94%) met the inclusion criteria. Sixteen subjects were excluded due to image artifacts (*n* = 15) or pathological findings (*n* = 1). The mean age of the analyzed subjects was 68 ± 14 years, with 178 men (65%) and 97 women (35%). Participants were categorized into three age groups: ≤60 years (*n* = 78, 28%), 61–75 years (*n* = 99, 36%), and >75 years (*n* = 98, 36%).

For T2 mapping, 200 examinations were performed, with 178 (89%) meeting the inclusion criteria. Twenty-one participants were excluded due to image artifacts, and one participant was excluded due to a pathological finding already identified in the T1 analysis. The mean age of the analyzed T2 group was 66 ± 16 years, with 125 men (70%) and 53 women (30%). The participants were categorized into three age groups: <60 years (*n* = 55, 31%), 60–75 years (*n* = 59, 33%), and >75 years (*n* = 64, 36%).

The general characteristics of the study population, including cardiovascular risk factors and comorbidities, are summarized in Table 1. Coronary artery disease (CAD) was present in 46% of participants, while arterial hypertension had the highest prevalence (59%). Dyslipidemia was detected in 37%, and diabetes mellitus was documented in 12%. Diastolic dysfunction was observed in 16%, and reduced left ventricular ejection fraction (LVEF < 55%) was diagnosed in 25%. Atrial fibrillation was present in 17% of participants, while chronic obstructive pulmonary disease (COPD) was documented in 7%. Among valvular heart diseases, tricuspid regurgitation was most common (13%), followed by aortic stenosis (5%). Less frequent findings included mitral stenosis (2%) and mitral regurgitation (2%).

### 3.2. Mean Values

#### 3.2.1. T1 Relaxation Time

A total of 275 measurements were conducted. The global mean T1 relaxation time was 1004.7 ± 49.8 ms [95%-CI: 998.8; 1010.6]. The mean values for the different myocardial layers were as follows: 1002.8 ± 68.5 ms in the basal layers (range: 885–1158 ms; 95%-CI: 988.8; 1016.8), 1001.1 ± 50 ms in the medial layers (range: 858–1191 ms; 95%-CI: 990.9; 1011.3), and 1010.1 ± 55.4 ms in the apical layers (range: 807–1187 ms; 95%-CI: 998.8; 1021.4).

#### 3.2.2. T2 Relaxation Time

A total of 178 measurements were included for T2 analysis. The global mean T2 relaxation time was 67.4 ± 8.6 ms [95%-CI: 66.1; 68.7]. The mean values for the myocardial layers were 64.5 ± 7.9 ms in the basal layers (range: 45.9–97.2 ms; 95%-CI: 62.5; 66.5), 67.5 ± 9.5 ms in the medial layers (range: 47.8–121.3 ms; 95%-CI: 65.1; 69.9), and 70.2 ± 8.6 ms in the apical layers (range: 47.1–108.1 ms; 95%-CI: 68.0; 72.4).

### 3.3. Intra- and Inter-Observer Variability

Intra- and inter-observer reproducibility of myocardial T1 and T2 measurements were assessed in a randomly selected subset of 30 examinations. Intra-observer variability was 6.8% for T1 (*p* = 0.01) and 9.1% for T2 (*p* = 0.02). Inter-observer variability was higher, with 10.2% for T1 (*p* = 0.02) and 12.8% for T2 (*p* = 0.03).

### 3.4. Influence of Age

#### 3.4.1. T1 Relaxation Time

**Figure 2:**
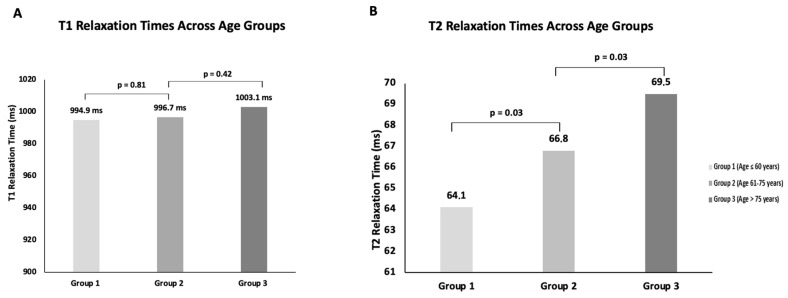
**Age-dependent distribution of myocardial relaxation times.** Mean values of myocardial relaxation times are shown for three age groups: Group 1 (≤60 years, light grey), Group 2 (61–75 years, medium grey), and Group 3 (>75 years, dark grey). (**A**) T1 relaxation times. (**B**) T2 relaxation times.

**Figure 3:**
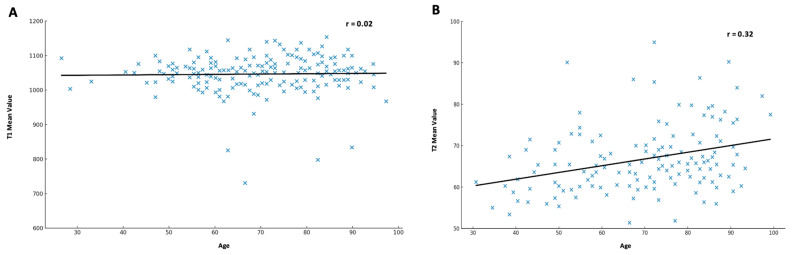
**Association between age and myocardial relaxation times.** Linear regression analysis of age and myocardial relaxation times. (**A**) No significant correlation was observed between age and T1 relaxation times (r = 0.02). (**B**) A moderate positive correlation was observed between age and T2 relaxation times (r = 0.32).

Subjects were categorized into three age groups: ≤60 years (*n* = 78, 28%), 61–75 years (*n* = 99, 36%), and >75 years (*n* = 98, 36%). The mean T1 relaxation times for these groups were 994.9 ± 46 ms, 996.7 ± 52.4 ms, and 1003.1 ± 60 ms, respectively. Statistical analysis showed no significant differences in T1 relaxation times between the age groups (*p* = 0.81; *p* = 0.42) (see Figure 2A). In addition, a linear regression analysis revealed no significant correlation between age and native myocardial T1 relaxation time, with a correlation coefficient of r = 0.02 (see Figure 3A).

#### 3.4.2. T2 Relaxation Time

For T2 mapping, participants were categorized into three age groups: <60 years (*n* = 55, 31%), 60–75 years (*n* = 59, 33%), and >75 years (*n* = 64, 36%). The mean T2 relaxation times for these groups were 64.1 ± 6.3 ms, 66.8 ± 6.9 ms, and 69.5 ± 6.7 ms, respectively. A significant difference in T2 relaxation times was observed between the age groups (*p* < 0.05), indicating an age-dependent increase in T2 values (see Figure 2B). This observation was further investigated using linear regression analysis, which demonstrated a statistically significant linear relationship between age and myocardial T2 relaxation time, with a correlation coefficient of r = 0.32 (see Figure 3B).

### 3.5. Influence of Gender

#### 3.5.1. T1 Relaxation Time

**Figure 4:**
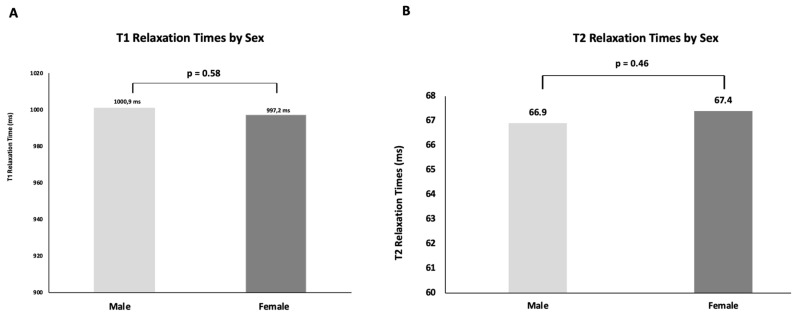
**Sex-dependent distribution of myocardial relaxation times.** Mean values of myocardial relaxation times are shown separately for male (light grey) and female (dark grey) patients. (**A**) T1 relaxation times. (**B**) T2 relaxation times.

The mean T1 relaxation time in women was 1000.9 ± 57.5 ms, while in men, it was 997.2 ± 51.3 ms. No statistically significant differences were observed between the two groups (*p* = 0.58) (see Figure 4A).

#### 3.5.2. T2 Relaxation Time

The mean T2 relaxation time was 67.4 ± 6.6 ms in women and 66.9 ± 6.7 ms in men. Statistical analysis showed no significant gender-based differences (*p* = 0.46) (see Figure 4B).

### 3.6. Influence of Comorbidities

#### 3.6.1. T1 Relaxation Time

Among the 275 subjects assessed for T1 mapping, 135 were diagnosed with CAD. The mean T1 relaxation times for subjects with single-vessel disease, two-vessel disease, and three-vessel disease were 1002.1 ± 37.5 ms, 1003.5 ± 30.1 ms, and 1003.0 ± 60.6 ms, respectively. No significant differences were observed between the CAD subgroups (*p* > 0.05).

Further analysis of comorbidities revealed that atrial fibrillation (AF) and mitral regurgitation significantly influenced T1 relaxation times. In AF patients (*n* = 50), the mean T1 relaxation time was 1012.2 ± 49.2 ms, which was significantly higher than in non-AF subjects (*p* < 0.05). Similarly, a significant increase in T1 relaxation time was observed in patients with mitral regurgitation (*p* < 0.05).

#### 3.6.2. T2 Relaxation Time

For T2 mapping, no significant differences in relaxation times were found among CAD subgroups (*p* > 0.05). However, an analysis of NYHA classification revealed significant variations in T2 values. Subjects with NYHA Class III–IV had higher T2 relaxation times compared to those in lower NYHA classes, indicating a potential association between myocardial tissue alterations and advanced heart failure stages (*p* < 0.05).

### 3.7. Summary of Findings

This study analyzed myocardial T1 and T2 relaxation times in a well-defined cohort, focusing on age, gender, and comorbidities as influencing factors. While no significant gender-based differences were found for either T1 or T2 values, age was a significant determinant of T2 relaxation time, with older participants exhibiting higher values (see Figure 3). Among comorbidities, atrial fibrillation and mitral regurgitation were associated with increased T1 relaxation times, while advanced NYHA stages showed a significant impact on T2 relaxation time. These findings underscore the necessity of standardized reference values for T1 and T2 mapping to improve diagnostic accuracy in clinical practice.

## 4. Discussion

Cardiovascular magnetic resonance (CMR) imaging has emerged as a crucial non-invasive tool for myocardial tissue characterization, particularly through the assessment of T1 and T2 relaxation times. This study aimed to establish normative reference values for myocardial T1 and T2 relaxation times in a real-world patient population and to investigate the influence of age, sex, and comorbidities on these parameters. Our findings provide significant insights into the interindividual and intraindividual variability of myocardial relaxation times and underscore the importance of standardizing these values for improved diagnostic accuracy.

### 4.1. T1 and T2 Relaxation Times in the Myocardium of a Representative Clinical Cohort 

The analysis of T1 relaxation times in our study demonstrated an average global T1 relaxation time of 1004.7 ms ± 49.8 ms, with specific values for the basal (1002.8 ± 68.5 ms), medial (1001.1 ± 50 ms), and apical myocardial layers (1010.1 ± 55.4 ms). These values are largely consistent with previously published data from studies conducted using 1.5 Tesla (T) MRI scanners [14,15]. However, differences between reported reference values highlight the influence of technical factors such as pulse sequence selection, scanner type, and population characteristics. Compared to studies conducted using 3T MRI systems, our study found a significant discrepancy in T1 values, particularly in the apical myocardial layers, which exhibited prolonged relaxation times at higher field strengths.

The observed variability underscores the need for scanner-specific reference values and harmonized imaging protocols to facilitate clinical translation. The range of T1 values (850 to 1178 ms) observed in our cohort further emphasizes interindividual differences, which may challenge the differentiation of normal physiological variation from early pathological changes. For instance, reported T1 values in patients with Fabry disease (853 ± 50 ms) and dilated cardiomyopathy (1017 ± 47 ms) overlap with our cohort’s values, indicating potential diagnostic ambiguities [16,17]. These findings reaffirm the critical role of T1 mapping in identifying subclinical myocardial abnormalities and highlight the potential of native T1 mapping as a valuable alternative for patients with contraindications to contrast agents.

### 4.2. Influence of Comorbidities on T1 Relaxation Times

Our study identified significant associations between T1 relaxation times and specific comorbidities, particularly atrial fibrillation (AF) and mitral valve regurgitation. Patients with AF exhibited significantly prolonged T1 relaxation times, reflecting the impact of chronic atrial remodeling, myocardial fibrosis, and altered hemodynamics on myocardial tissue composition [18]. The irregularity in heart rhythm and associated volumetric changes may contribute to increased myocardial stress and fibrosis, leading to prolonged relaxation times. This is consistent with previous findings demonstrating that AF is associated with an increased extracellular volume fraction and diffuse myocardial fibrosis, as evidenced by histological and imaging studies [19].

Partial volume effects can potentially influence both T1 and T2 mapping in patients with AF. In our study, we ensured high image quality and precise myocardial delineation, minimizing these effects. Although they cannot be entirely excluded, the consistent T2 measurements support the reliability of our findings. This approach is in line with previous studies, such as Zhao et al. (2016), highlighting the importance of optimizing imaging protocols to reduce artifacts in AF patients [20].

Similarly, mitral valve regurgitation was associated with prolonged T1 relaxation times, likely due to increased left ventricular (LV) workload and resultant myocardial remodeling. Chronic volume overload from mitral regurgitation has been shown to induce interstitial expansion and fibrosis, contributing to prolonged T1 relaxation times [21,22]. Future studies should further explore the correlation between the severity of valvular disease and myocardial T1 parameters to refine diagnostic thresholds for myocardial fibrosis.

Conversely, no significant associations were found between T1 relaxation times and other comorbidities, including coronary artery disease (CAD), diabetes mellitus, and chronic obstructive pulmonary disease (COPD). While previous research suggests that chronic metabolic and inflammatory disorders may contribute to subtle myocardial structural changes, our findings did not demonstrate statistically significant variations in T1 relaxation times in these patient groups [23]. This highlights the need for larger-scale studies to investigate whether subclinical myocardial involvement in these conditions is detectable using quantitative myocardial mapping techniques.

### 4.3. Age and Sex Influence on T1 Relaxation Times

Our data showed no significant sex-related differences in T1 relaxation times. The average T1 values for men (997.2 ± 51.3 ms) and women (1000.9 ± 57.5 ms) were comparable (*p* = 0.58), consistent with prior studies suggesting that sex has minimal impact on myocardial T1 properties [19]. However, some reports indicate that hormonal influences and differences in myocardial extracellular volume may contribute to subtle variations in T1 values, which warrants further investigation.

Age did not significantly influence T1 relaxation times in our study, as evidenced by comparable values across different age groups. This finding aligns with studies by Dabir et al., which also reported a lack of significant age-related differences in native T1 relaxation times [24]. However, trends toward prolonged T1 times in older individuals suggest potential age-related changes in myocardial tissue composition, including increased interstitial fibrosis and altered water content. Further studies utilizing histopathological correlation may help elucidate whether these changes reflect normal aging or early myocardial pathology.

### 4.4. T2 Relaxation Times and Their Determinants

The global T2 relaxation time in our study was 67.4 ms ± 8.6 ms, with basal (64.5 ms ± 7.9 ms), medial (67.5 ms ± 9.5 ms), and apical (70.2 ms ± 8.6 ms) myocardial layers demonstrating expected regional variation. These values are consistent with prior reports and highlight the inherent heterogeneity of myocardial T2 properties [13].

Unlike T1 relaxation times, T2 times showed a significant association with age. Older individuals exhibited prolonged T2 values, suggesting that aging may contribute to subtle changes in myocardial water content and interstitial properties [25]. This aligns with prior works, which reported age-related increases in T2 times in healthy individuals [25]. Such findings reinforce the need for age-adjusted reference values to avoid overdiagnosis of myocardial pathology in elderly patients.

In contrast to T1 mapping, no significant associations were observed between T2 relaxation times and comorbidities, including AF, mitral valve regurgitation, and CAD. The lack of correlation suggests that T2 mapping may be less sensitive to chronic fibrotic remodeling and more indicative of acute myocardial injury or edema [26]. Given the role of T2 mapping in detecting myocardial inflammation, future studies should explore its utility in differentiating acute from chronic myocardial processes across various disease states.

### 4.5. Generalizability of Results

While our findings establish robust reference values for T1 and T2 relaxation times at 1.5 Tesla using Philips platforms, caution is warranted when extrapolating these results to other MRI vendors and field strengths. Prior studies have demonstrated systematic differences in relaxation times across different vendors and between 1.5T and 3T systems, largely attributable to sequence design, magnet hardware, and reconstruction algorithms. Similarly, ethnicity has been identified as a potential source of physiological variation, with population-based studies suggesting modest, yet significant, differences in myocardial relaxation properties among diverse ethnic groups [15,27]. Consequently, our results should be interpreted within the technical and demographic context of the studied cohort, and multicenter efforts incorporating multiple vendors, field strengths, and ethnically diverse populations remain essential for establishing universally applicable normative values.

### 4.6. Clinical Implications and Future Directions

Our findings emphasize the necessity of standardizing myocardial relaxation time parameters to enhance the clinical utility of CMR. The establishment of reference values tailored to specific scanner settings and patient demographics will improve the accuracy of myocardial disease detection. Moreover, integrating AI into CMR image processing holds promise for automating myocardial segmentation, reducing observer variability, and improving diagnostic efficiency [28].

Further prospective studies with larger, multi-center cohorts are warranted to validate our findings and explore the prognostic significance of myocardial T1 and T2 alterations. Future research should also focus on refining imaging protocols to minimize technical variability and investigating the potential of novel contrast-free mapping techniques for broader clinical applicability.

## 5. Conclusions

This study provides a comprehensive assessment of myocardial T1 and T2 relaxation times in a representative clinical cohort. Our results confirm the robustness of myocardial mapping techniques while highlighting the influence of age and specific comorbidities on myocardial relaxation properties. The findings underscore the need for standardized reference values to optimize the diagnostic and prognostic utility of CMR-based tissue characterization. Future efforts should aim to integrate machine learning approaches for improved data analysis and establish normative databases that account for demographic and technical variations. Ultimately, advancing our understanding of myocardial relaxation properties will contribute to enhanced risk stratification and individualized treatment strategies for cardiovascular diseases.

## Figures and Tables

**Table 1:** **Baseline characteristics of patients with myocardial T1 (T1-Group) and T2 (T2-Group) relaxation time measurements.** Demographic and clinical characteristics, cardiovascular risk factors, and comorbidities of patients who underwent myocardial relaxation time assessment. Data are presented as mean ± standard deviation or as absolute numbers with percentages, where applicable.

	T1-Group *n* = 291 [in %]	T2-Group *n* = 200 [in %]	*p*-Value
Age [years]	70.8 [±14.4]	68.1 [±16.3]	0.056
Female sex	107 [37%]	60 [30%]	0.120
BMI > 30 kg/m^2^	32 [11%]	19 [10%]	0.968
Chronic Coronary Disease (CAD)	144 [49%]	92 [47%]	0.657
*Single-vessel disease (SVD)*	*24 [8%]*	*20 [10%]*	*0.440*
*Two-vessel disease (2VD)*	*30 [10%]*	*23 [12%]*	*0.594*
*Three-vessel disease (3VD)*	*83 [29%]*	*44 [22%]*	*0.152*
Chronic Kidney Disease (CKD)	27 [9%]	16 [8%]	0.696
Diastolisc Dysfunction	52 [18%]	39 [20%]	0.538
EF < 55%	72 [25%]	53 [27%]	0.526
Cardiomyopathy	17 [6%]	19 [10%]	0.104
Atrial Fibrilation (AF)	53 [18%]	28 [14%]	0.319
COPD	21 [7%]	13 [7%]	0.828
Hypertension	184 [63%]	117 [60%]	0.517
Nicotin usage	61 [21%]	36 [19%]	0.517
Dyslipoprotinemia	107 [37%]	72 [37%]	0.939
Diabetes Mellitus	35 [12%]	24 [12%]	0.910
NYHA Status			
*O*	*122 [42%]*	*81 [40%]*	*0.681*
*I*	*49 [17%]*	*38 [19%]*	*0.503*
*II*	*79 [27%]*	*55 [27%]*	*0.861*
*III*	*41 [14%]*	*24 [12%]*	*0.464*
*IV*	*1 [0%]*	*2 [1%]*	*0.319*
CCS Status			
*O*	*224 [77%]*	*158 [79%]*	0.674
*I*	*9 [3%]*	*10 [5%]*	*0.219*
*II*	*26 [9%]*	*12 [6%]*	*0.162*
*III*	*17 [6%]*	*6 [3%]*	*0.204*
*IV*	*12 [4%]*	*14 [7%]*	*0.243*
Severe Aortic Senosis	19 [7%]	14 [8%]	0.679
Severe Aortic Regurgitation	31 [11%]	31 [17%]	0.058
Severe Mitral Stenosis	7 [2%]	2 [1%]	0.298
Severe Mitral Regurgitation	63 [22%]	44 [24%]	0.615
Severe Tricuspid Regurgitation	38 [13%]	29 [16%]	0.442

## Data Availability

The datasets used and/or analyzed in the current study are available from the corresponding author on reasonable request.

## Abbreviations

AF	Atrial fibrillation
AI	Artificial intelligence
b-SSFP	Balanced steady-state free precession
CAD	Coronary Artery Disease
CCS	Canadian Cardiovascular Society
COPD	Chronic obstructive pulmonary disease
CMR	Cardiac magnetic resonance imaging
CVD	Cardiovascular disease
DGK	German Cardiac Society
EACVI	European Association for Cardiovascular Imaging
EPI	Echo planar imaging
ESC	European Society of Cardiology
GRaSE	Gradient and spin-echo sequence
ICD	Implantable cardioverter defibrillator
LGE	Late gadolinium enhancement
LVEF	Left ventricular ejection fraction
MOLLI	Modified Look-Locker Inversion Recovery
MR	Mitral regurgitation
NYHA	New York Heart Association
SCMR	Society for Cardiovascular Magnetic Resonance
TSE	Turbo spin echo
